# Circulating tumor DNA in B-cell lymphoma: technical advances, clinical applications, and perspectives for translational research

**DOI:** 10.1038/s41375-022-01618-w

**Published:** 2022-06-14

**Authors:** Eliza M. Lauer, Jurik Mutter, Florian Scherer

**Affiliations:** 1 Department of Medicine I, Medical Center – University of Freiburg, Faculty of Medicine, University of Freiburg, Freiburg, Germany; 2 Faculty of Biology, University of Freiburg, Freiburg, Germany; 3 German Cancer Consortium (DKTK) partner site Freiburg, German Cancer Research Center (DKFZ), Heidelberg, Germany

**Keywords:** Translational research, Risk factors

## Abstract

Noninvasive disease monitoring and risk stratification by circulating tumor DNA (ctDNA) profiling has become a potential novel strategy for patient management in B-cell lymphoma. Emerging innovative therapeutic options and an unprecedented growth in our understanding of biological and molecular factors underlying lymphoma heterogeneity have fundamentally increased the need for precision-based tools facilitating personalized and accurate disease profiling and quantification. By capturing the entire mutational landscape of tumors, ctDNA assessment has some decisive advantages over conventional tissue biopsies, which usually target only one single tumor site. Due to its non- or minimal-invasive nature, serial and repeated ctDNA profiling provides a real-time picture of the genetic composition and facilitates quantification of tumor burden any time during the course of the disease. In this review, we present a comprehensive overview of technologies used for ctDNA detection and genotyping in B-cell lymphoma, focusing on pre-analytical and technical requirements, the advantages and limitations of various approaches, and highlight recent advances around improving sensitivity and suppressing technical errors. We broadly review potential applications of ctDNA in clinical practice and for translational research by describing how ctDNA might enhance lymphoma subtype classification, treatment response assessment, outcome prediction, and monitoring of measurable residual disease. We finally discuss how ctDNA could be implemented in prospective clinical trials as a novel surrogate endpoint and be utilized as a decision-making tool to guide lymphoma treatment in the future.

## Introduction

With the advent of precision medicine, our understanding and knowledge of B-cell lymphoma biology, molecular subtypes, and genetic landscapes have substantially increased over the last decade [1–3]. Similarly, major recent advances in basic and translational research have enhanced therapeutic options in lymphoma, including novel targeted agents, bispecific monoclonal antibodies, and cellular-based immunotherapies such as the chimeric antigen receptor T- (CAR T-) cell therapy [4]. As a result, identifying patient subgroups with high risk for treatment failure, predicting clinical outcomes early at diagnosis or during therapy, and optimizing patient selection for specific treatment strategies have moved to the core of modern translational lymphoma research and patient management [5].

Invasive tissue biopsies are the gold standard to obtain molecular information and to stratify lymphoma patients into genetic subgroups [3, 6]. However, such invasive procedures have several limitations. Surgical interventions carry procedural risks and often cannot be performed in patients with severe pre-existing health conditions or when tumor lesions are inaccessible. Furthermore, tissue biopsies do not fully capture spatial and temporal tumor heterogeneity, because only one single tumor site is usually sampled, and serial biopsies are not available in most cases [7–12]. Therefore, precise and accurate technologies that facilitate detection, quantification, and characterization of B-cell lymphomas in real-time are needed to overcome these limitations and to help succeed novel strategies of lymphoma precision medicine.

**Fig. 1:**
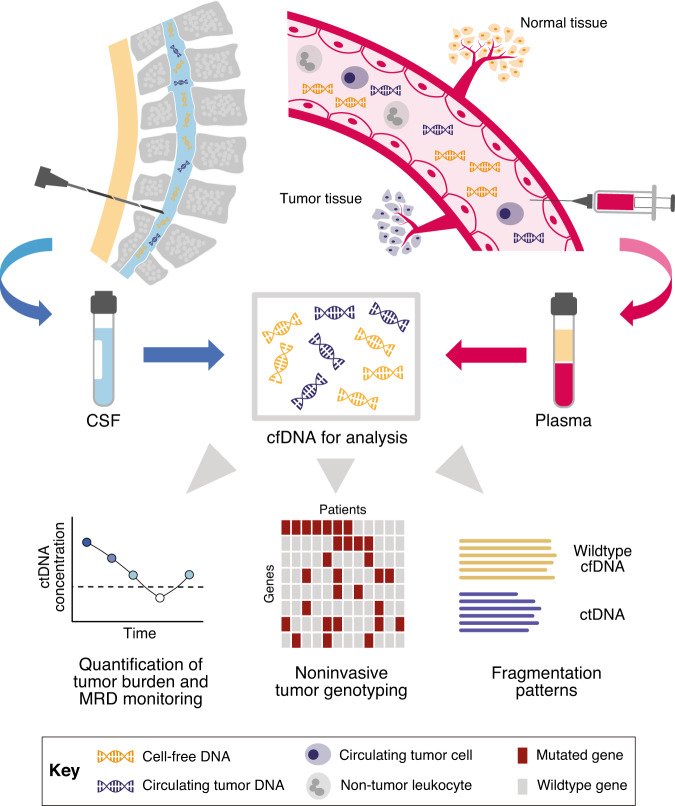
Sources of cell-free DNA and circulating tumor DNA in lymphomas and their different aspects as analytes for liquid biopsy. Cell-free DNA and circulating tumor DNA are released from malignant and non-malignant tissue into the blood stream and cerebrospinal fluid, where they can be accessed through blood draws and lumbar punctures. In lymphoma, ctDNA obtained from blood plasma or cerebrospinal fluid has been studied as a non- or minimal-invasive clinical biomarker. At the bottom, various aspects of cfDNA and ctDNA analyses in lymphoma patients are highlighted, including quantification of tumor burden and MRD monitoring during and after therapies, noninvasive tumor genotyping for lymphoma classification and characterization of tumor heterogeneity, and fragmentation patterns.

**Fig. 2:**
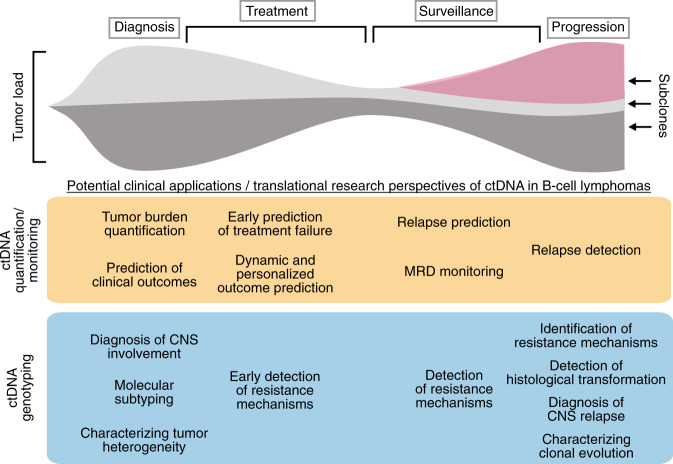
Potential clinical applications and perspectives for translational research of ctDNA in B-cell lymphomas. Profiling of ctDNA might be performed at various disease landmarks at diagnosis, during treatment, during surveillance, and at lymphoma progression to support decision-making by physicians or contribute novel insights to address innovative translational research questions. CNS central nervous system, MRD minimal residual disease.

‘Liquid biopsy’ has emerged as an innovative approach to detect and characterize cancers non- or minimal-invasively through profiling of tumor-derived analytes in body fluids, most commonly blood but also cerebrospinal fluid (CSF), urine, ascites, pleural fluid, or saliva [13–20]. Circulating tumor DNA (ctDNA) in the blood plasma or CSF has become the most investigated analyte in B-cell lymphomas, as the majority of lymphoma patients do not present with circulating disease and therefore, circulating tumor cells (CTCs) are usually a less attractive target (Fig. 1) [21]. Circulating tumor DNA is shed from tumor deposits into circulation and represents a subset of the total cell-free DNA (cfDNA) pool released from cells undergoing apoptosis or necrosis [22]. Major advances in polymerase chain reaction- (PCR-) and next-generation sequencing- (NGS-) based technologies have led to improved detection of minimal ctDNA amounts in body fluids, facilitating ultrasensitive detection of minute residual tumor masses during or after therapy (measurable residual disease, MRD) for early identification of treatment failure and prediction of disease relapse in numerous cancer entities including lymphoma. As ctDNA reflects all types of tumor-specific genetic alterations including single nucleotide variants (SNVs), translocations, insertions/deletions (indels), and copy number variations (CNVs), it potentially allows comprehensive assessment of spatial tumor heterogeneity between different tumor lesions, classification of molecular subtypes, and the identification of temporal heterogeneity such as the emergence of resistance mutations over time [11, 15]. Both non- or minimal-invasive quantification of tumor burden and the characterization of tumor heterogeneity have potential clinical utility at various lymphoma milestones, with MRD monitoring during and after treatment being the most established application of ctDNA as of yet (Fig. 2) [5, 21, 23–25].

In this review, we present an overview of current technologies used for ctDNA-based lymphoma quantification and profiling, illustrate advantages and limitations of the most commonly used liquid biopsy methods, discuss technical factors influencing their performance, and highlight recent advances to improve sensitivity and specificity. Furthermore, we highlight potential clinical applications and the perspectives for translational research of ctDNA analyses in various B-cell lymphoma entities at distinct clinical landmarks, and show how ctDNA could inform innovative clinical trials and guide personalized patient management in the near future.

## Technical considerations for ctDNA detection and quantification

The utility of liquid biopsy technologies largely relies on adequate sensitivity and specificity to detect minute amounts of ctDNA in body fluids. Recent developments in molecular biology, high-throughput analytics, and bioinformatics have overcome major obstacles such as pre-analytical limitations, low recovery rates of cfDNA molecules or the high abundance of technical errors introduced during library preparation and the hybridization process, and substantially enhanced the technical performance of liquid biopsy methods [21, 24, 26].

Several pre-analytical factors need to be considered to ensure optimal sample collection and cfDNA processing in lymphoma patients. First, the detection limit of most liquid biopsy technologies heavily depends on the number of cfDNA molecules analyzed. Concentrations of cfDNA in lymphoma are highly variable, ranging from a median of ~6.5 ng/mL of plasma in indolent follicular lymphoma (FL) to ~650 ng/mL in primary mediastinal B-cell lymphomas (PMBCL), and are significantly associated with tumor stage [27, 28]. Thus, at least 10 mL of blood (≈4–6 mL blood plasma) are generally recommended to obtain a sufficient number of cfDNA molecules for subsequent analyses [24, 28, 29]. Second, when blood is collected in EDTA tubes, plasma should be isolated within 6 hours to avoid contamination of the plasma fraction with cellular DNA released from peripheral blood mononuclear cell (PBMC) lysis [29]. Cell-stabilizing tubes such as Streck® or PAXgene® tubes minimize the contamination effect, are stable at room temperature for approximately 7 days, and are preferred if tubes need to be shipped or stored for a longer time [30]. Finally, the presence of germline variants and clonal hematopoiesis of indeterminate potential (CHIP) could act as confounders and hamper accurate detection of somatic tumor-derived aberrations in ctDNA, particularly in the setting of tumor-agnostic noninvasive genotyping. Thus, paired sequencing of germline DNA (either from plasma-depleted whole blood, saliva, or buccal swabs) and leukocyte-derived DNA is recommended to subtract those errors from cfDNA analysis [29, 31–34].

There are numerous technologies available for ctDNA profiling that can be categorized into PCR-based methods (e.g., allele-specific oligonucleotide PCR [ASO-PCR] and digital droplet PCR [ddPCR]) and NGS-based approaches (Table 1). They can further be divided into those technologies allowing sensitive MRD detection and those that facilitate both MRD quantification and comprehensive assessment of mutational landscapes (i.e., genotyping). While PCR assays are cost effective, relatively easy to use, and have a short turnaround time, they can only target one single or a small number of recurrent somatic variants and are therefore not the preferred choice for broad noninvasive genotyping. Similar to other single-gene methods, sensitivities of PCR-based methods usually do not exceed allele frequencies (AF) of ~0.005%, because the cfDNA input is typically limited to a maximum of ~20,000 haploid genome equivalents (hGE) per blood draw (Table 1) [35]. Therefore, they are frequently used for MRD monitoring in lymphomas with highly recurrent chromosomal translocations such as t(14;18) in FL or t(11;14) in mantle cell lymphoma (MCL) (preferentially ASO-PCR), or with stereotypic mutations such as *MYD88*^L265P^ in primary CNS lymphoma (PCNSL), waldenstrom macroglobulinemia (WM), or (MCD-, C5-) diffuse large B-cell lymphoma (DLBCL), and *EZH2*^Y641N^ in FL or (EZB-, C3-) DLBCL [3, 6].

**Table 1:** Comparison of liquid biopsy technologies for ctDNA genotyping and monitoring in lymphoma.

	Assay	Analytical sensitivity	ctDNA genotyping	ctDNA monitoring	Limitations
PCR-based	ASO-PCR	~0.005%	–	++	No multiplexing Limited input DNA
	ddPCR	~0.005%	–	++	Captures only one or a few markers Requires individual optimization Limited standardization Limited input DNA
NGS-based	IgHTS	~0.005%	–	++	Captures only one marker No standardized data interpretation Requires VDJ identification in tumor Limited input DNA
	Targeted HTS (e.g., CAPP-Seq)	~0.002%	+++	+++	Complex workflow Elaborate bioinformatics required No standardized data interpretation
	Duplex sequencing	~0.0002%	–	+++	Elaborate bioinformatics required No standardized data interpretation Limited recovery of duplex molecules
	PhasED-seq	~0.00005%	–	++++	Elaborate bioinformatics required No standardized data interpretation
	WES / WGS	~1%	+++	–	Low sensitivity Misses subclonal aberrations No standardized data interpretation Expensive

*ASO-PCR* allele-specific oligonucleotide polymerase chain reaction, *ddPCR* digital droplet PCR, *IgHTS* immunoglobulin high-throughput sequencing, *CAPP-Seq* Cancer Personalized Profiling by Deep Sequencing, *PhasED-seq* Phased Variant Enrichment and Detection Sequencing, *WES* whole-exome sequencing, *WGS* whole-genome sequencing.

NGS-based technologies allow massive parallel sequencing of DNA molecules in a single flow cell [35]. Lymphomas are characterized by patient-specific clonal rearrangements of their immunoglobulin (*Ig*) V(D)J regions (=clonotypes), which can be identified in tumor tissue and monitored in cfDNA over time by NGS-based methods utilizing universal primer sets targeting the *Ig* heavy and light chains (IgHTS, clonoSEQ®) (Table 1). This assay, provided by Adaptive Biotechnologies, is FDA approved for MRD detection in patients with chronic lymphocytic leukemia (CLL), multiple myeloma (MM), and B-cell acute lymphoblastic leukemia (B-ALL) and has been utilized in most lymphomas for disease monitoring [36–39]. However, like other single-gene assays, IgHTS only captures one single genetic marker and its ctDNA detection limit is restricted to the number of cfDNA molecules analyzed (typically ~0.005%, see above, Table 1). Furthermore, due to high rates of somatic hypermutation (SHM) in germinal center lymphomas, in particular FL and DLBCL, IgHTS fails to detect clonal V(D)J-rearrangements in ~20% of patients, which limits its applicability in these lymphoma entities [35, 37, 38, 40].

Targeted amplicon-based or hybrid-capture NGS technologies have several dedicated advantages over single gene assays (Table 1). They target hundreds of lymphoma-specific genetic regions and enable the identification of the entire spectrum of genetic alterations (i.e., SNVs, indels, translocations, and CNVs) [12, 21, 26, 27, 41–43]. They typically utilize entity-specific sequencing panels that cover genetic regions known to be frequently mutated in lymphomas. Consequently, they do not require patient-specific optimization, are usually applicable to a broader population of patients, allow comprehensive genotyping from cfDNA and, through the identification of subclonal and low-frequency alterations, facilitate characterization of spatial and temporal tumor heterogeneity. Due to the ability to track multiple mutations per patient simultaneously and recent major advances in molecular biology and in silico strategies that suppress technical errors, sensitivity of targeted NGS-based approaches could be substantially improved. For example, Cancer Personalized Profiling by Deep Sequencing (CAPP-Seq) combines a unique barcoding strategy with a downstream bioinformatics algorithm that largely eliminates sequencing errors and stereotypic background (integrated digital error suppression, iDES), facilitating ctDNA detection down to an AF of ~0.002% [12, 26, 27, 42].

In situations with extremely low tumor burden, particularly during treatment and at the end of lymphoma therapy, current methods are still limited by suboptimal sensitivity [44–46]. Prior studies have used somatic mutations identified on both DNA strands to further decrease ctDNA detection limits (‘duplex sequencing’). This NGS-based strategy reduces sequencing errors by requiring two concordant events on both Watson and Crick strands for the detection of one SNV, achieving an analytical sensitivity of ~0.0002% [26, 47, 48]. Yet, the majority of recovered DNA molecules are single-stranded and only a minority contains both strands. Since maximal recovery of hGE is essential to liquid biopsy NGS approaches, this inefficacy substantially limits the applicability of duplex sequencing in the real-world setting (Table 1) [21, 26, 49]. To overcome this limitation, an innovative approach has been developed that further maximizes analytical sensitivity and reduces background error rates by tracking two or more variants (‘phased variants’) on the same strand of one single DNA molecule (‘PhasED-seq’, Phased Variant Enrichment and Detection Sequencing). This method offers extremely low error profiles while maintaining high genome recovery, thus facilitating ctDNA monitoring down to an analytical detection limit of ~0.00005% (i.e., 1 in 2,000,000, Table 1). PhasED-seq seems particularly useful in B-cell lymphoma, as mutations accumulate in stereotyped genetic regions caused by ongoing SHM and aberrant SHM through the activity of the enzyme activation-induced cytidine deaminase (AID) [49–51]. A similar strategy has been introduced recently by *Meriranta* et al., providing additional evidence that tracking of phased variants can significantly improve sensitivity of ctDNA detection in lymphoma patients [52].

### Applications of ctDNA in lymphoma

#### Diagnostic tumor quantification by ctDNA and its prognostic value

The accurate reflection of tumor burden at diagnosis is a crucial characteristic of ctDNA as a noninvasive biomarker, because pretreatment disease burden is an established risk factor in various lymphoma entities and generally associated with worse outcomes. Numerous risk factors are a direct portrait of lymphoma burden such as the International Prognostic Index (IPI) in DLBCL, MCL, and FL [53–55], or total metabolic/radiographic tumor volume (TMTV/TRTV) from PET/CT scans or MRI [56]. Several research groups have investigated the relationship between pretreatment ctDNA concentrations and conventional markers of tumor burden and its role as a prognostic biomarker in B-cell lymphoma, as summarized in Table 2. In DLBCL, baseline ctDNA levels significantly correlate with IPI, TMTV as well as lactate dehydrogenase (LDH) concentrations and Ann Arbor stage [12, 27, 36, 39, 43, 52, 57, 58]. Importantly, this correlation can be directly translated into a prognostic effect, as pretreatment ctDNA concentrations have shown to be strongly predictive of clinical outcomes in univariate and multivariate analyses in patients receiving standard immunochemotherapy [12, 27, 52, 57, 58]. Furthermore, *Frank* et al. performed IgHTS on serial plasma samples from 69 patients with relapsed/refractory DLBCL (rrDLBCL) receiving anti-CD19 CAR T-cell therapy and demonstrated that pretreatment ctDNA levels significantly correlate with progression-free survival (PFS) and overall survival (OS) [39].

**Table 2:** Selected publications demonstrating clinical value of pretreatment ctDNA and cfDNA profiling in B-cell lymphomas.

	Author	PMID/doi	Parameter	Method	No. of patients	Correlation with			Prediction of	
						Radiographic tumor burden	Stage	Clinical score	PFS/EFS	OS
**DLBCL**	Roschewski et al.	25842160	ctDNA concentration	IgHTS	126	NA	I + II vs. III + IV *p* = 0.014	IPI *p* < 0.0001	NA	NA
	Scherer et al.	27831904	ctDNA concentration	Panel-directed NGS^a^	76	TMTV *p* < 0.0001, *r* = 0.67	I + II vs. III + IV *p* = 0.0003	IPI *p* < 0.0001	Continuous*p* = 0.008	Continuous *n.s.*
	Kurtz et al.	30125215	ctDNA concentration frontline	Panel-directed NGS^a^	217	TMTV *p* < 0.001	NA	IPI *p* < 0.001	< vs. > 2.5 log hGE/mL *p* = 0.007; Continuous *p* = 0.0008	< vs. > 2.5 log hGE/mL *n.s*.; Continuous *p* = 0.02
	Bohers et al.	30069017	ctDNA concentration	Panel-directed NGS	30	MTV low vs. high *p* < 0.001	*n.s*.	IPI *p* = 0.02	NA	NA
	Rivas-Delgado et al.	33122345	ctDNA concentration	Panel-directed NGS	79	TMTV *p* < 0.0001, *r* = 0.56	NA	IPI high vs. int-high *p* = 0.0018	Low vs. high *p* = 0.038; Continuous *p* = 0.0032	Low vs. high *p* = 0.007; Continuous *p* < 0.001
	Frank et al.	34133196	pre-LD ctDNA concentration	IgHTS	69	NA	I + II vs. III + IV *p* = 0.0196	IPI *p* = 0.0143	<10^1^ LG/mL vs. >10^3^ LG/mL *p* < 0.0001^b^	<10^1^ LG/mL vs. > 10^3^ LG/mL *p* < 0.0001^b^
	Alig et al.	33909455	ctDNA concentration	Panel-directed NGS^a^	267	TMTV *p* < 0.001	I + II vs. III + IV *p* < 0.001	IPI *p* < 0.001	< vs. > 2.5 log hGE/mL *p* < 0.001; Continuous *p* < 0.001	< vs. > 2.5 log hGE/mL *p* = 0.002; Continuous *p* < 0.001
	Meriranta et al.	34932792	ctDNA concentration	Panel-directed NGS	100	TMTV *p* = 5.2 × 10^−6^, *r* = 0.57	III vs. IV *p* = 0.00097	IPI *p* = 3 × 10^−4^	< vs. > 3.75 log hGE/mL *p* = 0.0037; Continuous *p* = 0.003	< vs. > 3.75 log hGE/mL *p* = 0.0024; Continuous *p* = 0.004
**HL**	Spina et al.	29449275	ctDNA concentration	Panel-directed NGS	80	NA	Ann Arbor *p* = 0.021 Lim. vs. Adv. *p* = 0.001	GHSG *p* = 0.013	NA	NA
	Sobesky et al.	10.1101/2021.03. 16.21253679	cfDNA concentration	Panel-directed NGS	111	NA	Ann Arbor *p* = 0.0001	IPS *p* < 0.0001	NA	NA
	Camus et al.	32079702	ctDNA concentration	Panel-directed NGS	60	MTV *p* < 0.001, *r* = 0.47	I + II vs. III + IV *p* = 0.002	IPS 0–2 vs. 3–5 *p* = 0.018	NA	NA
	Desch et al.	31431735	ctDNA concentration	Panel-directed NGS	72	MTV *p* = 0.0059, *r* = 0.33	Ann Arbor *n.s*.	NA	NA	NA
**MCL**	Lakhotia et al.	35143622	ctDNA concentration	IgHTS	48	TMTV *p* < 0.0001*, r* = 0.73	NA	MIPI *p* = 0.0094	< vs. > median *n.s*.; Continuous *p* = 0.03	< vs. > median *n.s*.; Continuous *p* = 0.01
	Agarwal et al.	30455436	ctDNA detection	Panel-directed NGS	23	NA	NA	NA	Det. vs. undet*. p* = 0.018	NA
**FL**	Sarkozy et al.	28060738	ctDNA concentration and detection	IgHTS	29	Bulky disease *n.s*.	NA	FLIPI *n.s*.	< vs. > median *p* = 0.004	NA
	Delfau-Larue et al.	29636326	cfDNA concentration	ddPCR	61	TMTV *p* < 0.0001, *r* = 0.6	NA	NA	High vs. low^c^ *p* = 0.04	NA
	Distler et al.	10.1182/ blood-2021-151096	ctDNA concentration	IgHTS	32	TMTV *p* < 0.006, *r* = 0.49	NA	NA	NA	NA
**CNSL**	Hattori et al.	29151258	ctDNA detection	ddPCR	14	NA	NA	NA	Det. vs. undet. *n.s*.	Det. vs. undet. *n.s*.
	Fontanilles et al.	28636991	cfDNA concentration ctDNA detection	Panel-directed NGS	25	TRTV *n.s*.	NA	NA	NA	< vs. > 64 ng/mL *n.s*.
	Mutter et al.	10.1182/ blood-2021-149644	ctDNA concentration and detection	Panel-directed NGS^d^	78	TRTV *p* < 0.001, *r* = 0.53	NA	MSKCC *n.s*.	Det. vs. undet. *p* < 0.001	Det. vs. undet. *p* = 0.002

When ‘Continuous’ is stated, the univariable result is shown. The comparison highlighted here shows the survival difference between the lowest and highest groups.

*PMID* Pubmed ID, *No.* number, *PFS* progression-free survival, *EFS* event-free survival, *OS* overall survival, *ctDNA* circulating tumor DNA, *cfDNA* cell-free DNA, *IgHTS* immunoglobulin high-throughput sequencing, *IPI* International Prognostic Index, *NGS* Next-generation sequencing, *hGE* haploid genome equivalents, *TMTV* total metabolic tumor volume, *MTV* metabolic tumor volume, *TRTV* total radiographic tumor volume, *Lim* limited, *Adv.* advanced, *GHSG* German Hodgkin Study Group risk score, *IPS* International Prognostic Score, *Det*. detected, *undet*. undetected, *vs.* versus, *LD* lymphodepletion, *LG* lymphoma genomes, *mL* milliliter, *ng* nanogram, *NA* not assessed, *n.s.* not significant, *DLBCL* diffuse large B-cell lymphoma, *HL* Hodgkin lymphoma, *FL* follicular lymphoma, *MCL* mantle cell lymphoma, *CNSL* central nervous system lymphoma.

^a^CAPP-Seq.

^b^ctDNA levels were categorized into four groups.

^c^Threshold of 2550 equivalent genomes per mL of plasma.

^d^PhasED-seq.

Similar results have been reported for other lymphomas. For example, both the amount of cfDNA and ctDNA concentrations at baseline assessed by targeted NGS-assays correlated with TMTV, stage, and clinical risk scores such as the International Prognostic Score (IPS) in HL patients [59–61]. *Desch* et al. used a targeted-capture HTS approach that covered 87 distinct genes to profile and quantify ctDNA in pediatric HL [62]. They found significant associations of ctDNA levels with TMTV and bulky disease, while ctDNA did not reflect disease stage in this cohort of patients [62]. In patients with MCL, ctDNA measured by IgHTS accurately mirrored radiographic tumor burden and ctDNA positivity assessed either by IgHTS or targeted-capture HTS was strongly predictive of clinical outcomes [25, 63, 64]. Lymphoma types with an indolent growth pattern such as FL or in lymphomas that are confined to the central nervous system (i.e., PCNSL or isolated secondary CNSL [iSCNSL]), ctDNA levels in blood plasma are substantially lower and ctDNA detection is more challenging [28, 65–67]. Here, the association between ctDNA levels and tumor burden or clinical outcomes is not fully established. For example, while previous studies failed to show any correlation in PCNSL [65, 66], *Mutter* et al. recently demonstrated that ctDNA mirrors MRI tumor volumes and predicts PFS and OS, both in log-rank and cox regression analyses, using PhasED-seq for ctDNA quantification [68]. In FL, three studies targeting either ctDNA clonotypes by IgHTS or assessing cfDNA concentrations by ddPCR revealed significant correlations with baseline TMTV and showed that higher ctDNA/cfDNA levels were associated with shorter PFS [38, 69, 70] (Table 2).

#### ctDNA-based response assessment during treatment

Treatment responses are highly variable in patients with B-cell lymphomas and accurate prediction of treatment failure or clinical outcomes would substantially improve personalized therapeutic strategies. While interim-PET/CT-guided prognostication and treatment has been implemented in standard clinical care of patients with HL [71], conventional methods for risk stratification and personalized treatment selection are limited in non-Hodgkin lymphomas. For example, the IPI, TMTV measured by PET/CT, or cell of origin (COO) subtypes have largely failed to demonstrate any utility for directing treatment in DLBCL [12, 72–81]. Therefore, ctDNA assessment at distinct time points during treatment and at the end of therapy might help overcome these limitations and improve patient risk stratification.

Concentrations of ctDNA usually change rapidly during lymphoma treatment. Quantitative response assessment of on-treatment ctDNA has shown to be highly prognostic in various lymphoma entities, as summarized in Table 3. In an early study by *Roschewski* et al., the authors observed that ctDNA negativity by IgHTS after two cycles of dose-adjusted EPOCH ± rituximab was associated with a favorable five-year PFS in patients with DLBCL [36]. Similarly, another study by *Kurtz* et al. defined two landmarks for the assessment of ‘molecular response’ during standard immunochemotherapy in DLBCL patients, utilizing serial ctDNA monitoring by CAPP-Seq: a 2-log reduction in ctDNA after 1 cycle of therapy (=early molecular response, EMR) and a 2.5-log reduction in ctDNA after 2 cycles of therapy (=major molecular response, MMR) [21, 27]. Patients achieving EMR and MMR had significantly superior event-free survival (EFS) and OS than patients without a 2- or 2.5-log ctDNA drop, both in frontline and salvage therapeutic settings [27]. However, EMR and MMR still misclassified certain patients at these fixed time points. Thus, the authors developed an approach integrating various prognostic factors measured before and during treatment (i.e., IPI, pretreatment ctDNA, COO, EMR, MMR, and interim PET/CT) into one single algorithm that updates the patient’s risk dynamically over time as more information becomes available (Continuous Individualized Risk Index, CIRI) [82]. This personalized method was applied to an independent validation cohort and outperformed conventional risk factors such as IPI, COO, interim PET/CT, and even EMR and MMR, for outcome prediction [82].

**Table 3:** Selected publications demonstrating prognostic value of on-treatment ctDNA monitoring in B-cell lymphomas.

	Author	PMID/doi	Parameter	Method	No. of patients	Prediction of PFS / EFS	Prediction of OS
**DLBCL**	Roschewski et al.	25842160	ctDNA det. vs. undet. after c2	IgHTS	108	TTP *p* < 0.0001	*n.s*.
	Kurtz et al.	30125215	2-log drop of ctDNA at c2d1	Panel-directed NGS^a^	Validation set 1:73	*p* = 0.015	*p* < 0.001
			2.5-log drop of ctDNA at c3d1			*p* < 0.001	*p* = 0.0047
	Kurtz et al.	34294911	ctDNA det. vs. undet. at c3d1^b^	Panel-directed NGS^c^	52	*p* = 0.0047	NA
	Merryman et al.	10.1182/blood-2020-140965	ctDNA det. vs. undet. in ASC sample	IgHTS	97	*p* < 0.001	*p* = 0.037
	Frank et al.	34133196	ctDNA det. vs. undet. at d21 after CAR T-cell therapy^d^	IgHTS	57	*p* < 0.0001	*p* = 0.0012
	Assouline et al.	27166360	ctDNA increase vs. decrease at d15	Panel-directed NGS, ddPCR	25	*p* = 0.0049	*p* = 0.00117
	Meriranta et al.	34932792	ctDNA det. vs. undet. after 3 cycles	Panel-directed NGS	58	*n.s*.	*n.s*.
**HL**	Spina et al.	29449275	2-log drop of ctDNA after 2 cycles	Panel-directed NGS	24	*p* < 0.001	*p* = 0.004
**MCL**	Lakhotia et al.	35143622	ctDNA det. vs. undet. after c1 of induction	IgHTS	48	*p* = 0.002	*n.s*.
			ctDNA det. vs. undet. after c2 of induction		50	*p* = 0.005	*p* = 0.03
	Smith et al.	10.1182/blood-2019-129323	ctDNA det. vs. undet. after c3 of induction	IgHTS	164	*p* = 0.002	NA
**CNSL**	Mutter et al.	10.1182/ blood-2021-149644	ctDNA detection during induction therapy	Panel-directed NGS^d^	49	*p* = 0.0002	*p* = 0.004

*PMID* Pubmed ID, *No.* Number, *PFS* progression-free survival, *EF*S event-free survival, *OS* overall survival, *ctDNA* circulating tumor DNA, *IgHTS* immunoglobulin high-throughput sequencing, *NGS* next-generation sequencing, *ddPCR* digital droplet polymerase chain reaction, *CAR* chimeric antigen receptor, *det.* detected, *undet.* undetected, *vs.* versus, *c* cycle, *d* day, *TTP* time to progression, *AS*C apharesis stem cell, *NA* not assessed, *n.s.* not significant, *DLBCL* diffuse large B-cell lymphoma, *HL* Hodgkin lymphoma, *MCL* mantle cell lymphoma, *CNSL* central nervous system lymphoma.

^a^CAPP-Seq.

^b^ctDNA assessed by PhasED-seq in DLBCL patients who were ctDNA negative by CAPP-Seq after 2 cycles.

^c^PhasED-seq.

^d^Prognostic value of ctDNA was assessed at multiple time points after CAR T-cell therapy, including day 21, 28, and 56.

The value of ctDNA for risk assessment in DLBCL was further shown in other therapeutic settings. For example, CAR T-cell therapy has emerged as a novel strategy to treat B-cell lymphomas and has introduced new challenges for risk stratification and response assessment [25]. Two major recent studies evaluated the role of ctDNA in DLBCL patients who were treated with CAR T-cell therapy in more detail. *Frank* et al. used IgHTS to detect V(D)J clonotypes in the plasma of 72 relapsed/refractory (rr) DLBCL patients undergoing treatment with axicabtagene ciloleucel (axi-cel). They found that 70% of patients responding to CAR T-cell therapy had undetectable ctDNA 7 days after infusion, compared to 13% of progressing patients. At multiple time points after axi-cel infusion (days 21, 28, and 56), ctDNA positivity was predictive of clinical outcomes, both for PFS and OS [39]. In another study, *Sworder* et al. used CAPP-Seq to profile ctDNA at various landmarks before and after axi-cel therapy. They demonstrated that ctDNA levels were prognostic for PFS in univariate analyses both at diagnosis and at several time points after CAR T-cell infusion [83]. Similarly, *Merryman* et al. applied IgHTS to 141 patients with rrDLBCL undergoing autologous stem cell transplantation (autoSCT) and found that the identification of ctDNA in apheresis stem cell samples was predictive of PFS and OS [46]. Another study showed that an increase of ctDNA at day 15 of panobinostat treatment in rrDLBCL patients was significantly associated with treatment failure [84].

Beyond DLBCL, studies have demonstrated accurate risk assessment by ctDNA quantification during treatment in various lymphoma types. In PCNSL, ctDNA positivity assessed by PhasED-seq during curative-intent induction therapy strongly predicted clinical outcomes, both PFS and OS [85]. In HL, *Spina* et al. found that a 2-log reduction of ctDNA after 2 cycles of ABVD was associated with favorable PFS and OS [59]. One other study highlighted that both ctDNA positivity and levels of ctDNA assessed by targeted NGS correlated with PET/CT Deauville scores after 2 cycles of therapy [60]. In a large cohort of pediatric patients with HL, *Desch* et al. explored whether ctDNA detection during treatment correlated with radiographic response assessment by PET/CT. They found that ctDNA was not detectable in 43 patients (0/43) showing favorable PET results (i.e., qPET <3), while 5 out of 6 patients with unfavorable PET/CT (i.e., qPET >3) were ctDNA-positive [62]. In MCL, patients receiving dose-adjusted R-EPOCH plus bortezomib had favorable clinical outcomes when ctDNA was undetectable by IgHTS after 1 or 2 cycles of therapy or at the end of induction treatment [64]. Similarly, *Smith* et al. presented data on ctDNA evaluation after 3 cycles of bendamustine-based induction therapy (IgHTS) and observed shorter PFS in MCL patients with positive ctDNA [86] (Table 3).

#### ctDNA as a biomarker for MRD detection after therapy and during surveillance

After treatment, lymphomas are typically monitored by radiographic imaging, including CT scans or PET/CT. Yet, their utility is controversial due to suboptimal specificity, and serial scans are no longer recommended for routine lymphoma surveillance [87–89]. In contrast, ctDNA as a biomarker is usually more disease-specific and allows noninvasive and serial monitoring without radiation exposure. MRD monitoring and lymphoma surveillance after completion of therapy is certainly the most established application of ctDNA. Its prognostic value and the role of ctDNA for relapse prediction/detection have been explored in various publications (Table 4). Three independent studies demonstrated that serial monitoring of DLBCL patients in complete remission (CR) either by IgHTS or CAPP-Seq facilitates the detection of lymphoma recurrence in the vast majority of cases, with a ~3–6 month lead time prior to radiographic imaging [12, 36, 37]. However, *Kumar* et al. recently reported a moderate ctDNA detection rate of 56% at or before clinical relapse in a prospective multicenter trial assessing the performance of ctDNA monitoring after DLBCL frontline treatment, applying the single gene assay IgHTS [44]. In other therapeutic settings, *Frank* et al. recently showed robust detection of ctDNA either before or at radiographic relapse in 29 of 30 (94%) DLBCL patients with initial response to CAR T-cell therapy [39]. *Merryman* et al. demonstrated high ctDNA detection rates by IgHTS in relapsing patients following high-dose chemotherapy and autoSCT [46]. In MCL, IgHTS identified ctDNA during surveillance or at disease progression in 62% of patients, with a median lead time of 7.2 months [64].

**Table 4:** Selected publications highlighting the clinical utility of ctDNA as a biomarker after therapy and during surveillance in B-cell lymphomas.

	Author	PMID/doi	Parameter	Method	No. of patients	Prediction of PFS / EFS	Prediction of OS	Other findings
**DLBCL**	Roschewski et al.	25842160	Relapse detection by ctDNA during surveillance	IgHTS	107	NA	NA	ctDNA detection in 88% of relapses, median lead time of 3.5 months
	Scherer et al.	27831904	ctDNA det. vs. undet. during surveillance	Panel-directed NGS^a^	25	*p* = 0.0003	*n.s*.	ctDNA detection in 100% of relapses, mean lead time of 2 months
	Kumar et al.	10.1182/blood-2020-138889	Relapse detection by ctDNA during surveillance	IgHTS	43	NA	NA	ctDNA detection in 56% of relapses
	Merryman et al.	10.1182/blood-2020-140965	ctDNA detection post ASCT	IgHTS	20^b^	NA	NA	ctDNA detection in 90% of relapses
	Frank et al.	34133196	Relapse detection by ctDNA after CAR T-cell therapy	IgHTS	30	NA	NA	ctDNA detection in 94% of relapses
	Kurtz et al.	34294911	ctDNA det. vs. undet. end of treatment	Panel-directed NGS^c^	19	*p* = 2.7 × 10^−6^	NA	NA
	Meriranta et al.	34932792	ctDNA det. vs. undet. end of treatment	Panel-directed NGS	71	*p* = 0.00098	*p* = 0.0049	NA
**HL**	Camus et al.	27479820	*XPO1* E571K det. in ctDNA end of treatment	dPCR	28	*n.s*. (0.091)	*n.s*. (0.0601)	NA
**MCL**	Lakhotia et al.	35143622	ctDNA det. vs. undet. end of induction	IgHTS	50	*p* = 0.003	*p* = 0.02	NA
			Relapse detection by ctDNA during surveillance		40	NA	NA	ctDNA detection in 62% of relapse, median lead time of 7.2 months
	Kumar et al.	33558202	Relapse detection by ctDNA/CTC during surveillance	IgHTS	9	NA	NA	ctDNA detection in 67% of relapse

*PMID* Pubmed ID, *No*. number, *PFS* progression-free survival, *EFS* event-free survival, *OS* overall survival, *ctDNA* circulating tumor DNA,* IgHTS* immunoglobulin high-throughput sequencing, *NGS* next-generation sequencing, *det*. detected, *undet.* undetected, *vs.* versus, *c*, cycle, *d*, day, *TTP* time to progression, *ASCT* autologous stem cell transplantation, *CTC* circulating tumor cell, *NA* not assessed, *n.s.* not significant, *DLBCL* diffuse large B-cell lymphoma, *HL* Hodgkin lymphoma, *MCL* mantle cell lymphoma.

^a^CAPP-Seq.

^b^20 patients who relapsed and had at least 1 plasma sample available within 100 days of relapse.

^c^PhasED-seq.

Several other studies explored the prognostic value of ctDNA as a landmark after the end of treatment, a situation in which MRD detection is particularly challenging due to soberingly low amounts of ctDNA in blood plasma. In DLBCL, three studies reported favorable clinical outcomes in patients with undetectable ctDNA after completion of therapy using targeted-capture NGS-based technologies [12, 49, 52]. In MCL, *Lakhotia* et al. showed that ctDNA detection by IgHTS after induction therapy is associated with shorter PFS and OS [64] (Table 4).

Finally, the utility of MRD monitoring in FL through liquid biopsy technologies has been extensively investigated in a plethora of publications. However, data in FL mainly rely on the assessment of CTCs in peripheral blood (PB) and bone marrow (BM), not ctDNA or cfDNA. Therefore, these studies are not covered in this review and we encourage readers to consult other dedicated sources such as *Pott* et al. [90].

#### ctDNA for lymphoma diagnosis and noninvasive genotyping

Histopathological assessment of lymphoma tissue obtained from invasive surgical procedures is the gold standard for lymphoma diagnosis, characterization of genetic landscapes, and subtype classification. Yet, noninvasive genotyping by ctDNA profiling from body fluids might represent a complementary tool in certain situations in which tumor tissue is inaccessible or cannot be obtained repeatedly over the course of the disease. The current clinical relevance of noninvasive tumor genotyping by ctDNA is certainly lower than the utility of MRD monitoring and tumor quantification, which are closer to translation. However, noninvasive profiling of tumor genotypes could have important implications as a precision medicine tool in the future, but its potential for clinical translation will largely depend on the development of subtype-specific targeted therapies for treatment selection [91]. For example, FL and DLBCL patients carrying *EZH2* mutations identified in lymphoma tissue or ctDNA show a better response to the EZH2 inhibitor tazemetostat than patients with wildtype *EZH2* [92, 93]. Furthermore, the identification of co-existing mutations in *MYD88* and *CD79B* might help identify DLBCL patients who are most likely to respond to the BTK inhibitor ibrutinib [94]. Similarly, mutations in *MYD88, CCND1* or in genes involved in the NFκB pathway appear to be associated with resistance to ibrutinib in MCL [24, 95]. Here, noninvasive genotyping from ctDNA might help guiding treatment decisions in a subset of cases where tumor tissue is unavailable.

A basic requirement of tumor-agnostic noninvasive genotyping is that liquid biopsy robustly mirrors tumor mutational patterns. Numerous studies have shown that the concordance between tissue-based and ctDNA-based genotyping is usually greater than 70%, even if the tumor cell content is exceptionally low like in HL (0.1–3%) [12, 41, 57, 59–62]. In general, mutation detection rates from tumors are higher than from plasma specimens due to the larger tumor content. However, mutations present in plasma but not in tumor tissue are frequently observed, indicating that ctDNA profiling can capture variants that are missed by single-site tumor biopsies (i.e., spatial heterogeneity) [12, 41, 57, 59]. For example, plasma genotyping by targeted NGS identified mutations in *CARD11* and *PIM1* genes in a patient diagnosed with FL that were not detected in a diagnostic inguinal lymph node biopsy. Yet, these mutations were shared between plasma and the patient’s transformed FL biopsy of a retroperitoneal mass 9 months later at disease progression, indicating that both indolent and aggressive clones were already present before histological transformation (HT) [12]. Other publications demonstrated simultaneous capturing of two *EZH2-*mutated clones in plasma by ddPCR that originate from two distinct tumor locations of a patient with FL, or the occurrence of *XPO1* mutations in 29% of HL patients who did not show any *XPO1* aberrations in tumor biopsies [96, 97]. *Camus* et al. provide additional evidence that genetic profiling from ctDNA in HL patients could have some decisive advantages over tumor genotyping due to low tumor cell content in this disease. They found that 52% of all mutations detected in 42 HL patients at diagnosis were exclusively present in plasma but not in corresponding tumor specimens [61].

Tumor biopsies can be particularly challenging in patients with central nervous system lymphoma (CNSL). Here, stereotactic serial biopsies are the gold standard to obtain brain tumor material for subsequent histopathological evaluation. However, invasive neurosurgical biopsies can be inconclusive or delayed due to concurrent steroid treatment and carry procedural risks, especially in patients with deep brainlesions [98–102]. Therefore, non- or minimal-invasive ctDNA profiling from plasma or CSF seems desirable in this group of patients, especially because flow cytometry and cytopathology from CSF are insensitive and require large sample volumes [103]. Several studies demonstrated moderate to high detection rates of ctDNA in CSF or plasma of PCNSL patients by utilizing either PCR-based methods for *MYD88*^L265P^ detection or broader NGS-based technologies, suggesting that a subset of patients with suspected brain lymphoma might be able to forego invasive surgical procedures [20, 68, 104–110]. Another major clinical challenge is the occurrence of CNS relapses in DLBCL patients and the inability to detect occult CNS involvement by conventional CSF flow cytometry and cytopathology. *Olszewski* et al. were able to detect ctDNA in CSF by IgHTS in 8 out of 19 (42%) DLBCL patients with high risk of CNS involvement but no overt CNS disease. Importantly, no patients with negative ctDNA but 29% with detectable ctDNA developed CNS relapse one year after DLBCL diagnosis [111].

Intravascular large B-cell lymphoma (IVLBCL) is another rare DLBCL subtype characterized by lymphoma cells infiltrating blood vessels but no obvious tumor mass, making conventional diagnosis by imaging or tissue biopsies extremely challenging [5, 24, 112]. Interestingly, variant allele frequencies and the number of identified mutations seem to be higher in ctDNA compared to biopsy-derived DNA in this disease [113]. *Shimada* et al. applied whole exome sequencing (WES) to comprehensively profile ctDNA in 18 IVLBCL patients. They found an enrichment of mutations associated with ABC-DLBCL (i.e., mutations in *MYD88* and *CD79B*) and frequent rearrangements involving programmed cell death ligands 1 and 2 (*PD-L1/PD-L2*) [114].

#### ctDNA for assessment of tumor heterogeneity

In DLBCL, mutational landscapes are highly heterogenous and allow classification of patients into subgroups that have significant implications for clinical outcomes [3, 6]. DLBCL tumors can further be classified according to their transcriptionally distinct B-cell differentiation state (COO: germinal center B cell-like (GCB) and activated B cell-like (ABC) DLBCL [1, 115–118]. Technologies used to evaluate COO from tumor tissue such as the gold standard gene-expression profiling (GEP) or immunohistochemistry algorithms are either unavailable in clinical routine or limited due to suboptimal classification performance [2]. Previous reports have shown that noninvasive classification of DLBCL tumors according to their COO phenotypes is feasible, either based on the mutational landscape of ctDNA or fragmentation patterns of cfDNA [12, 119]. The latter approach uses promotor fragmentation entropy and targeted deep sequencing of transcription start sites to infer expression of genes of interest and classify histological and molecular subtypes in DLBCL [119, 120].

The mutational landscapes of relapsed lymphomas are often substantially different from diagnostic tumor specimens [8, 121]. While serial tissue biopsies at lymphoma relapse are often not performed, ctDNA genotyping might add important information on molecular tumor heterogeneity over time. Indeed, three previous studies demonstrated clonal divergence between the diagnostic tumor specimen and plasma ctDNA at lymphoma relapse in DLBCL, FL, and HL patients using targeted-capture NGS [12, 41, 59]. Interestingly, while genomic divergence was relatively moderate in rrDLBCL and FL, the greatest molecular distance was observed in FL patients undergoing histological transformation (tFL), reflecting the biological shift from an indolent to an aggressive behavior [12]. Histological transformation of indolent lymphoma entities to aggressive lymphomas occurs at a rate of 2–3% per year and is associated with an unfavorable prognosis [122, 123]. Tumor biopsies at transformation are often not performed or fail to detect the transformed tumor site [25, 124]. By incorporating the magnitude of the genomic distance and the amount of ctDNA in plasma within a mathematical model, HT could be predicted noninvasively by CAPP-Seq prior to clinical detection with high sensitivity and specificity [12, 28].

In many cases, the emergence of novel subclones over time reflects a process of clonal selection under treatment pressure, especially with targeted agents. Various studies revealed that molecular mechanisms of resistance in lymphoma can be captured noninvasively by ctDNA profiling. For example, *Agarwal* et al. demonstrated robust detection of emerging resistance alterations in blood plasma of MCL patients receiving ibrutinib plus venetoclax within a phase II clinical trial, using a targeted amplicon-based NGS sequencing panel (42 genes) and low-coverage WGS. Among these acquired genetic events were the loss of chromosome 9p21.1-p23.4 and mutations in components of the SWI-SNF chromatin-remodeling complex [63, 125]. Other studies in DLBCL demonstrated the emergence of resistance mutations by serial ctDNA genotyping using targeted NGS panels, including novel *BTK*^C481S^ resistance mutations in patients receiving ibrutinib monotherapy. [12, 41, 52, 126]. Most recently, *Sworder* et al. observed the emergence of mutations in *CD19, PAX5*, and *TP53* following DLBCL relapse after CAR T-cell therapy, representing candidate resistance mechanisms to this novel therapeutic approach [83]. Finally, *Spina* et al. systematically explored mutational evolution patterns in serial ctDNA samples from 13 HL patients with lymphoma relapse following ABVD frontline therapy or salvage treatment. While they found that ancestral mutations mostly persist over time, all cases demonstrated clonal shifts with novel mutations detected in plasma at lymphoma recurrence such as *PIM1, IRF8*, or *TNFAIP3* [59].

### Future directions and conclusions

Circulating tumor DNA has emerged as an attractive biomarker in B-cell lymphomas with various potential clinical applications. Yet, there are multiple aspects and dimensions of ctDNA analyses, each of which revealing different facets of a patient’s lymphoma. The value of ctDNA for tumor quantification, MRD monitoring, and risk stratification has been extensively explored over recent years, in part due to tremendous advances of innovative technologies that facilitate ultrasensitive ctDNA detection. Thus, this application can be considered closest to translation, with prospective clinical trials warranted to investigate whether early ctDNA profiling on-treatment or MRD monitoring during surveillance in lymphoma lead to improved outcomes and toxicity profiles. For example, patients who show insufficient molecular response based on ctDNA quantification could benefit from alternative therapeutic strategies, ideally at a time point where disease burden is lowest to increase the efficacy of salvage regimens or novel strategies such as CAR T-cell therapy [127–129]. On the other hand, patients with favorable ctDNA responses might do just as well with reduced cycles of chemotherapy or less toxic agents. In general, ctDNA has the potential to serve as a novel surrogate endpoint to help accelerate and improve clinical trial designs and drug development.

Other aspects of ctDNA in lymphoma such as noninvasive tumor genotyping, characterizing tumor heterogeneity or fragmentation patterns are more exploratory but are increasingly relevant for addressing important translational research questions and could be leveraged to overcome limitations of tissue biopsies. For example, serial assessment of ctDNA could enhance our understanding of molecular factors underlying clonal evolution and treatment resistance in lymphoma, particularly in lymphoma types where tumors are largely inaccessible such as CNS lymphomas. Noninvasive detection of resistance mechanisms might also facilitate treatment modifications right before patients undergo clinically overt lymphoma progression. Furthermore, cfDNA fragmentation patterns or methylation features could have potential utility for lymphoma classification and characterizing molecular and histological subtypes, augmenting standard pathological procedures.

However, the lack of standardization and harmonization of liquid biopsy technologies between laboratories currently hamper the broad implementation of ctDNA profiling across countries and in multicenter clinical trials. Yet, several initiatives were launched to define standards for pre-analytical handling, panel design, assay performance, and bioinformatics, including the ‘ctDNA working group meeting at the ASH Annual Meeting’ and the ‘15-ICML workshop on ctDNA’ in 2019 [29].

Ultimately, with the combination of recent major technical advances allowing the detection of vanishing amounts of ctDNA and novel machine learning approaches that facilitate intelligent implementation of the various dimensions of ctDNA as an analyte, we envision the prospective evaluation of the clinical value of ctDNA for lymphoma genotyping, risk stratification, and MRD monitoring in the near future.
